# Can genetic assignment tests provide insight on the influence of captive egression on the epizootiology of chronic wasting disease?

**DOI:** 10.1111/eva.12895

**Published:** 2019-12-09

**Authors:** William L. Miller, W. David Walter

**Affiliations:** ^1^ Pennsylvania Cooperative Fish and Wildlife Research Unit Department of Ecosystem Science and Management Intercollege Graduate Degree Program in Ecology The Pennsylvania State University University Park PA USA; ^2^ U.S. Geological Survey Pennsylvania Cooperative Fish and Wildlife Research Unit The Pennsylvania State University University Park PA USA

**Keywords:** admixture, captive egression, chronic wasting disease, genetic assignment tests, *Odocoileus virginianus*

## Abstract

Identifying the sources of ongoing and novel disease outbreaks is critical for understanding the diffusion of epizootic diseases. Identifying infection sources is difficult when few physical differences separate individuals with different origins. Genetic assignment procedures show great promise for assessing transmission dynamics in such situations. Here, we use genetic assignment tests to determine the source of chronic wasting disease infections in free‐ranging white‐tailed deer (*Odocoileus virginianus*) populations. Natural dispersal is thought to facilitate the geographic diffusion of chronic wasting disease, but egression from captive cervid populations represents an alternative source of infection that is difficult to detect due to physical similarities with wild deer. Simulated reference populations were created based on allele frequencies from 1,912 empirical microsatellite genotypes collected in four sampling subregions and five captive facilities. These reference populations were used to assess the likelihood of ancestry and assignment of 1,861 free‐ranging deer (1,834 noninfected and 27 infected) and 51 captive individuals to captive or wild populations. The ancestry (*Q*) and assignment scores (*A*) for free‐ranging deer to wild populations were high (average *Q*_wild_ = 0.913 and average *A*_wild_ = 0.951, respectively), but varied among subregions (*Q*_wild_ = 0.800–0.947, *A*_wild_ = 0.857–0.976). These findings suggest that captive egression and admixture are rare, but risk may not be spatially uniform. Ancestry and assignment scores for two free‐ranging deer with chronic wasting disease sampled in an area where chronic wasting disease was previously unobserved in free‐ranging herds indicated a higher likelihood of assignment and proportion of ancestry attributable to captive populations. While we cannot directly assign these individuals to infected facilities, these findings suggest that rare egression events may influence the epizootiology of chronic wasting disease in free‐ranging populations. Continued disease surveillance and genetic analyses may further elucidate the relative disease risk attributable to captive and wild sources.

## 1 INTRODUCTION

The geographic distribution and diffusion of wildlife diseases are commonly related to the probability of contact among infected and susceptible individuals or populations (Ostfeld, Glass, & Keesing, 2005). Because of this, disease management strategies often focus on minimizing contact rates between infected groups and populations that are most at risk (Wobeser, 2002). These strategies are predicated on the ability to identify the putative source of novel infections accurately. Spatial epidemiological models have become an important tool for evaluating diffusion patterns associated with contact patterns in wild populations (Hefley, Hooten, Russell, Walsh, & Powell, 2017). Predicting transmission patterns for epizootic diseases with multiple sources of potential infections is likely to be more difficult when there are few physical differences among potential sources. Developing methods to evaluate the contribution and risk of alternative sources of infection is likely to improve the response and management of epizootic diseases in natural populations.

Disease spillover from captively reared populations is a potential alternative source of infection that may contribute to novel or ongoing outbreaks in sympatric wildlife populations (Nituch, Bowman, Beauclerc, & Schulte‐Hostedde, 2011). Captive populations can experience higher prevalence and infection rates when compared to their free‐ranging counterparts, indicating that they may act as important disease reservoirs (Keane et al., 2008). Management practices, such as electrical or double fencing, can be employed to reduce interactions among captive and wild populations (VerCauteren, Lavelle, Seward, Fischer, & Phillips, 2007a, 2007b). Despite this, captive egression, defined here as the intentional or unintentional release of captively reared individuals into wild populations, may still occur. For example, Kidd, Bowman, Lesbarrères, and Schulte‐Hostedde (2009) found a high proportion of mink (*Neovision neovision*) sampled in close proximity to captive mink facilities were either likely escapes or recent ancestors of escapees. This may contribute to the higher disease prevalence rates observed in wild populations near these facilities (Nituch et al., 2011). While captive egression is recognized as a potential mechanism for disease establishment and transmission in areas where captive and wild populations occur in sympatry, its contribution to outbreaks in wild populations is often overlooked. This may be due, in part, to the difficulty of discriminating between captive and wild individuals based on physical characteristics.

Despite physical similarities, captive and wild individuals can often be distinguished using molecular genetic techniques (Witzenberger & Hochkirch, 2014). Genetic assignment tests can be used to estimate an individual's ancestry composition and to directly assign individuals to a population of origin (Pritchard, Stephens, & Donnelly, 2000; Rannala & Mountain, 1997). These analyses can offer insights into the origination of disease cases when the source is unknown (Remais, Xiao, Akullian, Qiu, & Blair, 2011). While assignment tests can be useful for identifying the putative source of novel infections, they can be prone to certain types of error that affect conclusions made about pathogen spread. Genetic assignment methods may be biased in cases where potential source populations are not sampled (Manel, Gaggiotti, & Waples, 2005), which can be common when using samples collected from disease surveillance. Disease surveillance sampling is often skewed toward sources or areas where samples can be easily accessed and obtained (Nusser, Clark, Otis, & Huang, 2008). This may lead to incorrect assignments or biased coancestry estimates if an individual originated from an unsampled or undersampled population since there is no adequate reference for that source. Additionally, variance in sample sizes among populations can skew ancestry estimates and lead to spurious inference regarding origin (Wang, 2017).

The recent introduction of assignment methods based on simulated reference populations may help to alleviate these sources of bias. In this framework, reference populations are composed of simulated genotypes, created conditionally on the allele frequencies of empirical samples from putative source categories (e.g., captive and wild samples; Karlsson, Diserud, Moen, & Hindar, 2014). Because allele frequencies are reflective of a mixture of empirical sampling units, these simulated reference populations can be used to capture and represent the among‐sample variability in these broader categories. Therefore, simulated reference populations are likely a better generalization of source categories in cases where individuals may have sourced from unsampled populations and may have increased power to detect migrants and admixture when compared to assignment procedures based solely on the clustering of empirical genotypes (White, Miller, Dowell, Bartron, & Wagner, 2018). Further, reference populations can also be used to standardize estimates of ancestry by creating simulated populations of equal sample size and uniform mixture, thereby minimizing the probability of spurious inference related to unbalanced sampling schemes (Karlsson, Diserud, Moen, & Hindar, 2014).

### 1.1 Chronic wasting disease

Chronic wasting disease is a fatal, transmissible spongiform encephalopathy of white‐tailed deer (*Odocoileus virginianus*) and other cervids caused by a misfolded isoform of the prion protein (Williams, Miller, Kreeger, Kahn, & Thorne, 2002). Chronic wasting disease has been found in free‐ranging and captive cervid populations in 24 U.S. States and three Canadian provinces in North America (Carlson et al., 2018), free‐ranging and managed herds in Scandinavia (Benestad, Mitchell, Simmons, Ytrehus & Vikøren, 2016; Vikøren et al., 2019), and captive populations in South Korea (Kim et al., 2005; Sohn et al., 2002). Demographic models predict that chronic wasting disease may lead to population declines (Edmunds et al., 2016), and the effects may be sustained since prions can remain infectious for long periods in contaminated environments (Miller, Williams, Hobbs, & Wolfe, 2004). Because cervids are critical members of trophic systems where they occur, the continued spread of chronic wasting disease has been identified as a threat to biodiversity conservation efforts (Sutherland et al., 2018). Cervids are also an important cultural and economic resource, and chronic wasting disease can affect the economic benefit provided to human communities (Bishop, 2004). Thus, there is a strong imperative to identify the potential source of novel outbreaks so that mitigation practices can be targeted effectively to slow the geographic diffusion of the disease across landscapes.

Some cervids have been domesticated and are kept in captive herds. So, while natural dispersal is thought to be a driver of the geographic diffusion of chronic wasting disease in free‐ranging populations (Green, Manjerovic, Mateus‐Pinilla, & Novakofski, 2014; Hefley et al., 2017), interactions with infected captive individuals represent an alternative source of infection where captive and wild herds are kept in sympatry. The relative risk that captive herds pose to the establishment and spread of chronic wasting disease is a topic of considerable debate among wildlife professionals (Schuler, Wetterau, Bunting, & Mohammed, 2016). Translocation of infected individuals has been described as a possible mechanism for spreading the disease to new regions. For example, the movement of infected elk from South Dakota contributed to the establishment of chronic wasting disease in Canada (Kahn, Dubé, Bates, & Balachandran, 2004) and then subsequently to South Korea (Kim et al., 2005; Sohn et al., 2002). These examples highlight the risk of disease establishment associated with long‐range translocations, but the extensive contact networks maintained among proximal captive herds may also increase local transmission risk (Carrollo, 2016; Rorres et al., 2017). A recent study from Pennsylvania, a state with over 1,000 captive cervid herds, found that more than 50 percent of herds in the region have participated in the transfer of at least one deer between facilities (Rorres et al., 2017). Such translocations, combined with the intentional or unintentional release of infected individuals, may pose a risk to adjacent free‐ranging populations and facilitate the spread of chronic wasting disease into novel areas (Gerhold & Hickling, 2016). Captive cervid facilities often maintain specialized barriers to limit contact among captive and wild populations (VerCauteren et al., 2007a, 2007b). Management agencies will also quarantine infected herds to minimize the spread of chronic wasting disease among captive facilities (Carrollo, 2016). Despite this, chronic wasting disease is commonly detected in free‐ranging populations in close proximity to infected captive herds following establishment (Adams, Murphy, & Ross, 2016), indicating a possible relationship between captive egression and the establishment of new disease foci. There has been previous documentation of infected deer escaping from captive facilities (Joly et al., 2003), but the link between captive egression and disease outbreaks in free‐ranging populations is largely speculative.

### 1.2 Objectives

Evaluating the extent of captive egression may provide important insights regarding the epizootiology of chronic wasting disease where free‐ranging and captive populations co‐occur. Here, we utilized genetic assignment algorithms based on simulated reference populations to explore competing scenarios regarding the emergence of chronic wasting disease. Microsatellite genotypes collected from free‐ranging white‐tailed deer and nearby captive cervid facilities in the mid‐Atlantic region of the United States were used to estimate ancestry coefficients and assignment scores to captive and wild genetic clusters in an area of recent infection. We evaluated the background rate of captive ancestry and assignment in free‐ranging populations in order to determine the relative extent of egression. The following specific scenarios regarding origin were also assessed for 27 cases of chronic wasting disease: (1) The infected individual had a potential origin from the free‐ranging population, (2) the infected individual had a potential origin from a captive deer herd, or (3) the infected individual potentially shares ancestry with a captive herd.

## 2 Methods

### 2.1 Study area and sample collection

**Figure 1:**
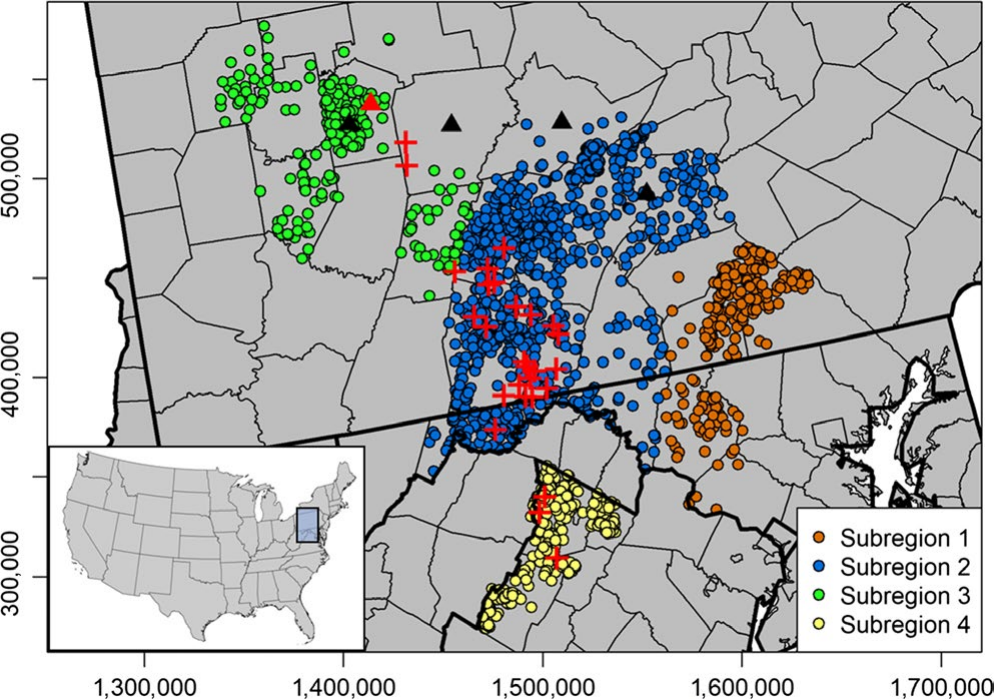
The distribution of 1,861 samples collected from free‐ranging white‐tailed deer in the mid‐Atlantic region of the United States. Samples were grouped into four distinct subregions that generally coincide with disease management units and ecophysiographic provinces (Piedmont = subregion 1, Ridge‐and‐Valley = subregions 2 and 4, Allegheny Plateau = subregion 3). Samples infected with chronic wasting disease are indicated by red crosses (*n* = 27). Locations of captive cervid facilities were mapped to county centroids in order to maintain anonymity. Four facilities where no disease was detected are represented by black triangles and one infected facility is represented by a red triangle

Samples were collected from an area in the mid‐Atlantic region of the United States with known disease incidence (Figure 1). Region‐wide prevalence rates were previously estimated to be ≤1% (Evans, Kirchgessner, Eyler, Ryan, & Walter, 2016; Evans, Schuler, & Walter, 2014); however, novel cases have been found in the free‐ranging population outside of the core management areas. Chronic wasting disease has also been found in captive facilities in Pennsylvania near outbreaks occurring in free‐ranging deer. Samples were collected from counties within and proximal to disease surveillance areas. These counties were grouped into four subregions (Figure 1), which generally approximate disease management efforts in the region and three ecophysiographic provinces separated by topographic escarpments (Piedmont = subregion 1, Ridge‐and‐Valley = subregions 2 and 4, and Allegheny Plateau = subregion 3). Disease management areas in subregions 2 and 4 were established in response to the detection of chronic wasting disease in free‐ranging populations from these regions; however, cases have recently been found in captive facilities in southern Pennsylvania. Disease management areas in subregions 1 and 3 were initially established due to the detection of chronic wasting disease in captive herds, and novel cases were also detected recently in free‐ranging populations in subregion 3 (Figure 1). These deer were approximately 50 km from the local disease focus in subregion 2 but were located in close proximity to captive facilities infected with chronic wasting disease (Figure 1).

We collected a total of 1,861 samples from free‐ranging, white‐tailed deer populations throughout the region (1,834 uninfected and 27 infected; Figure 1). Samples from free‐ranging populations consisted of muscle or connective tissue biopsies and were collected in conjunction with disease surveillance efforts and as part of a concurrent study at the Pennsylvania State University. A total of 50 samples were collected from five captive herds (Figure 1). Muscle biopsies were collected from one captive herd with known incidence of chronic wasting disease and two additional captive facilities in subregion 3. Blood samples were collected from two facilities in subregion 2, both of which provided DNA samples voluntarily. An additional deer identified by agency biologists as a likely captive escape, based on ear tags found on the animal, was found in free‐ranging populations in northeastern Pennsylvania and included in the captive pool. While two additional deer from subregion 3 were also found with ear tags, we could not exclude these two samples from the wild pool because public testimony indicated that these were captured as fawns and tagged.

### 2.2 Genetic analysis

Genomic DNA was extracted using the Qiagen DNeasy Blood and Tissue extraction kits (Qiagen). DNA from both blood and tissue samples was extracted using the manufacturer's protocol for each. The following modifications were made to the extraction protocol for tissue samples: (1) Tissue digestions were incubated for at least four hours to ensure samples were completely lysed; (2) DNA elutions were carried out with a single 150 µl volume in order to maximize DNA concentration. The following modifications were made to the extraction protocol for blood samples: (1) DNA elutions were carried out with a single 100 µl volume in order to maximize DNA concentration; and (2) a second wash step was performed using the DNA elution in order to maximize DNA yield. All samples were genotyped using a panel of 11 microsatellite markers that were shown to be polymorphic and amplify consistently in the focal populations (Miller, Edson, Pietrandrea, Miller‐Butterworth, & Walter, 2019). We reanalyzed a subset of the data to ensure reproducibility of results, estimated the percentage of missing data, and checked all loci for null alleles, deviations from Hardy–Weinberg equilibrium, and genetic linkage (Appendix S1).

### 2.3 Creation of reference populations

Reference populations were created following the general methods of Karlsson et al. (2014). The program HybridLab (version 1.0; Nielsen, Bach, & Kotlicki, 2006) was used to generate simulated reference populations consisting of 500 individuals each. Individuals that were missing genotypes at one or more loci were not included in the generation of reference populations. A single wild reference population was created to represent free‐ranging deer conditional on the allele frequencies of the four sampling subregions. While we did not test for population substructure within and among these four subregions and did not consider them separate populations in assignment analyses, we did maintain this stratification so that the wild reference population incorporated samples throughout the geographic extent of the study region. An equally sized subset of deer was randomly drawn from each of the four subregions, corresponding to the lowest sample size among the four (*n* = 230; Table 1). Free‐ranging deer known to be infected with chronic wasting disease were excluded from simulation. For captive populations, we chose to use all captive individuals in simulations, despite variance in sample size among herds and regardless of disease status, because all captive populations were characterized by small sample sizes (*n* = 1–16). While these sample sizes were small, the majority of captive cervid facilities in this region maintain stocking densities of less than 20 individuals (Rorres et al., 2017). Thus, these sample sizes are likely representative of genetic patterns among these and similar captive facilities. One captive deer was excluded from the generation of the reference cluster because pedigree records indicated that it was a wild deer that was legally brought into captivity following human habituation. We ensured that patterns of genetic diversity were similar between sampling units and simulated reference clusters by calculating several descriptive genetic statistics using GenAlEx (version 6.503; Peakall & Smouse, 2006, 2012) and Geneclass2 (version 2.0; Piry et al., 2004). These included the average number of alleles per locus (*N*_A_), Nei's unbiased expected heterozygosity (*H*_E_), and the number of private alleles relative to the wild sample (*P*_A_). We also ensured that all reference populations were appropriate representations of among‐sample genetic variance using a principal coordinate analysis based on *F*_ST_ estimates in GenAlEx (version 6.503; Peakall & Smouse, 2006, 2012). Reference populations were considered adequate representations of the genetic variation observed among captive and wild sampling units if they were located centrally in ordination space within the cluster of empirical populations that they were created to represent. Reference clusters would be considered biased if their position was skewed toward a particular sampling unit and not centroidal. Because sample sizes varied among sampling localities, we recognize that simulated populations may not be exact centroids in ordination space. Instead, we ensured that these clusters were generally approximate by visual inspection. We did not include one of the captive facilities (due to small sample size; *n* = 2), or the free‐ranging deer from northeastern Pennsylvania that was presumed to be captive in the principal component analysis. We also tested for deviations from Hardy–Weinberg expectations using exact tests in Genepop (version 4.6; Raymond & Rousset, 1995) for both simulated reference populations.

**Table 1:** Sample sizes (*n*), genetic summary statistics (*N*_A_ = average number of alleles per locus, *H*_E_ = Nei's unbiased estimated of heterozygosity, and *P*_A_ = number of private alleles), average ancestry to the wild cluster (*Q_wild_*), and average assignment score to the wild cluster (*A_wild_*) for wild and captive white‐tailed deer sampled in the mid‐Atlantic region of the United States

Samples	*n*	*N*_A_	*H*_E_	*P*_A_	*Q*_wild_	*A*_wild_
Wild	1,834 (500)	15.46 (15.09)	0.853 (0.859)	–	0.913	0.951
Subregion 1	230 (*230)*	13.18 *(13.18)*	0.841 *(0.841)*	1^a^ (*1)*	0.910	0.938
Subregion 2	1,092 (*230)*	14.73 *(14.00)*	0.845 *(0.851)*	2^a^ (*1)*	0.929	0.967
Subregion 3	231 (*230)*	13.82 *(13.73)*	0.840 *(0.840)*	1^a^ *(1)*	0.800	0.857
Subregion 4	281 (*230)*	14.36 *(14.27)*	0.842 *(0.842)*	4^a^ *(4)*	0.947	0.976
Captive	50 (500)	10.82 (10.82)	0.810 (0.800)	0^b^ (0)	0.077	0.059

Note

Sample sizes and summary statistics for the wild and captive reference populations are displayed in parentheses. Sample sizes and summary statistics for subregions used to create the wild reference population are italicized.

a Private alleles for each subregion are reported in relation to the others. Value in parentheses indicates number remaining in the sample used in simulation.

b Private alleles for captive herds are reported in relation to the wild population. Value in parentheses indicates number remaining in the sample used in simulation.

### 2.4 Ancestry analysis

Simulated reference clusters were used to evaluate the ancestry of empirical samples. The Bayesian algorithm implemented in STRUCTURE (version 2.3.4) was used to estimate the coefficient of ancestry (*Q*; Pritchard et al., 2000), which in this case was used to assess the proportion of an individual's genetic ancestry that could be attributed to a wild or captive origin. Independent STRUCTURE analyses were carried out for each empirical genotype with 500 simulated genotypes from each reference populations included in STRUCTURE analyses for a total of 1,001 genotypes. Simulated populations served as a standardized reference for coancestry estimates for each STRUCTURE analysis. All analyses were conducted using 100,000 Markov Chain Monte Carlo repetitions after a burn‐in period of 50,000 steps. Each individual was tested separately against reference populations to minimize biases related to variations in sample size among clusters. Two populations (*K* = 2) were assumed for all tests corresponding to the dichotomy between captive and wild populations. Since *K* was defined a priori and was not estimated by the algorithm, only a single iteration was performed for each test. STRUCTURE analyses were performed in parallel using the ParallelStructure R package (version 1.0; Besnier & Glover, 2013). Because ancestry proportions for each cluster are inversely proportional for a *K* = 2, we report ancestry estimates based on the proportion attributed to a wild origin (*Q*_wild_). Therefore, individuals with low *Q*_wild_ have a higher likelihood of sharing ancestry with captive populations.

### 2.5 Population assignment

We used the Geneclass2 (version 2.0; Piry et al., 2004) to estimate assignment scores (*A*) and probabilities (*p*) for each individual. In both cases, the simulated captive and wild reference clusters were defined as “reference populations” and all empirical samples were defined as “samples to be assigned.” Assignment scores were calculated using the Rannala & Mountain (1997) method. Because assignment scores are also inversely proportional when using two reference populations, we report assignment scores based on the likelihood of assignment to wild origin (*A*_wild_). We tested the hypotheses of wild and captive origin by estimating assignment probabilities for each individual based on the methods of Paetkau, Slade, Burden, and Estoup (2004). Assignment probabilities were calculated using 10,000 Monte Carlo simulations of the reference populations. We rejected the hypotheses of captive and wild origin with an *α* = 0.05. Free‐ranging deer where we failed to reject the hypothesis of captive origin were identified as potentially egressed individuals.

## 3 RESULTS

### 3.1 Creation of reference populations

Wild deer had higher allelic diversity (*N*_A_ = 15.46) and higher rates of heterozygosity (*H*_E_ = 0.853) than captive deer (*N*_A_ = 10.82; *H*_E_ = 0.810). Few private alleles were observed among subregions (*P*_A_ = 1–4), and no private alleles were observed in captive samples or samples with chronic wasting disease. Patterns of genetic diversity were similar between simulated reference clusters and the empirical samples they were used to represent (Table 1). Private alleles included in the wild reference cluster occurred in low frequencies (<1%) and were not expected to influence assignment analyses.

**Figure 2:**
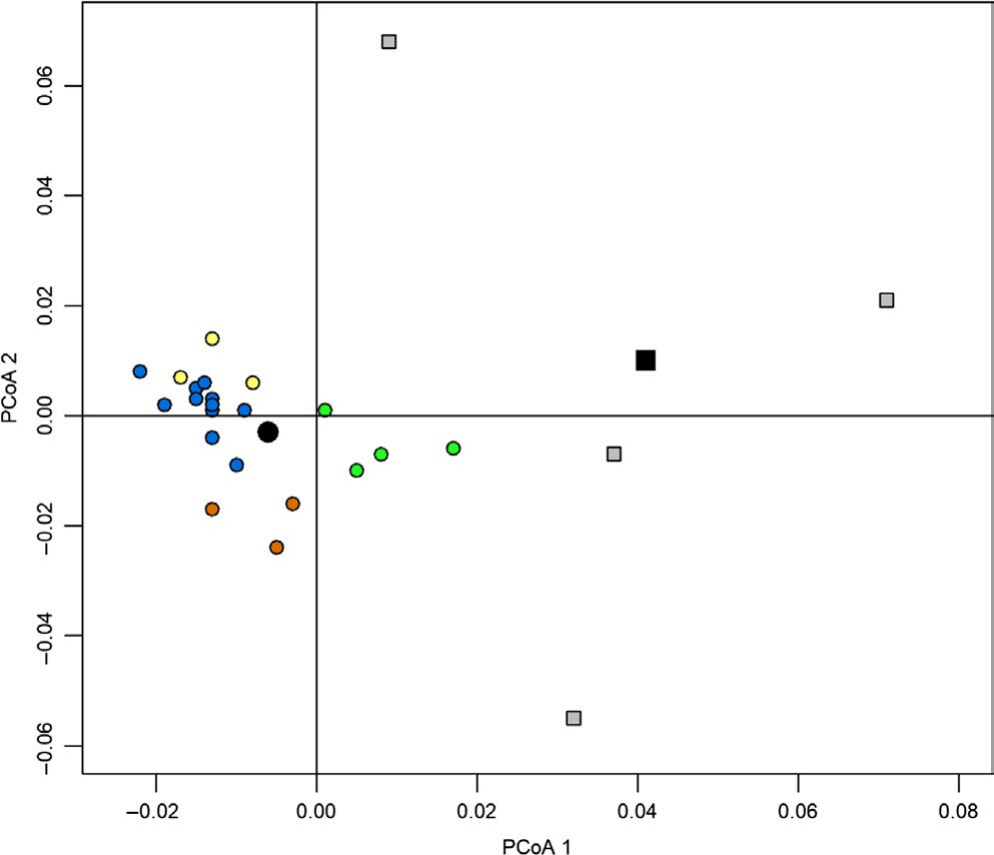
Principal coordinate analysis based on genetic distances (*F*_ST_) among free‐ranging (circles) and captive (squares) white‐tailed deer sampling units. Free‐ranging samples are subset by counties within each of the four subregions (orange = subregion 1, blue = subregion 2, green = subregion 3, yellow = subregion 4). Captive samples are subset by individual captive facilities. Simulated wild and captive reference populations used in genetic assignment tests are represented by black symbols

Genetic differentiation between captive and wild sampling units was low overall (*F*_ST_ = 0.003–0.091, mean = 0.023). The greatest degree of allelic divergence occurred among captive sampling units (mean *F*_ST_ = 0.064), but there was also greater differentiation between captive and wild sampling units than among wild sampling units (mean *F*_ST_ of 0.042 and 0.015, respectively). Wild sampling units clustered tightly in ordination space and were more genetically similar, while captive populations tended to be more variable among facilities (Figure 2). Despite differences in variability, the principal coordinate analysis indicates that captive and wild sampling units were separated in ordination space along axis 1 (21.83% of the variance explained; Figure 2). Reference populations were approximately centroidal for both wild and captive clusters. Given the separation between wild and captive sample clusters and the centrality of simulated reference populations within those clusters, we concluded that the simulated populations were adequate in summarizing the average degree of genetic divergence among these clusters and could be used as references in assignment tests. All loci conformed to Hardy–Weinberg expectations for each reference population following a Bonferroni correction for multiple comparisons (*p* > .0023).

### 3.2 Ancestry analysis

**Figure 3:**
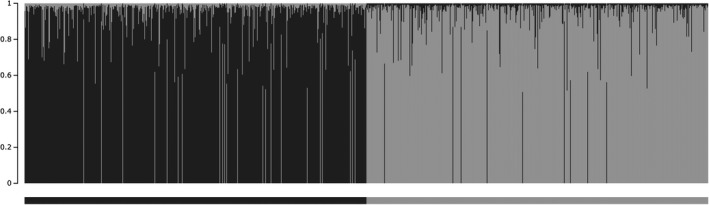
STRUCTURE bar plot representing the individual ancestry coefficients (vertical bars) for simulated reference populations with two clusters designated (*K* = 2). The horizontal bars indicate the 500 samples included in the wild (dark gray) and captive (light gray) reference populations. This figure was created using Structure Plot (version 2.0; Ramasamy, Ramasamy, Bindroo, & Naik, 2014)

**Figure 4:**
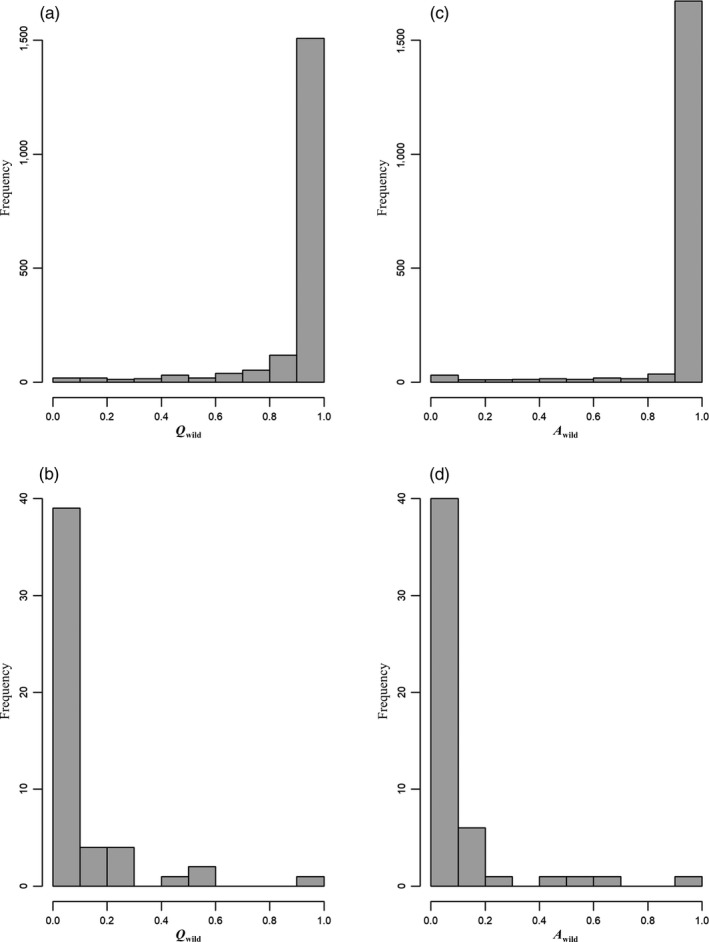
The distribution of ancestry scores for (a) 1,834 free‐ranging and (b) 51 captive white‐tailed deer from the mid‐Atlantic region of the United States. The distribution of assignment scores for (c) 1,834 free‐ranging and (d) 51 captive white‐tailed deer from the mid‐Atlantic region of the United States

We were able to distinguish graphically between the wild and captive reference populations in STRUCTURE (Figure 3). To evaluate the stability of ancestry coefficient estimates, we measured the average standard deviation of *Q*_wild_ estimates for the 1,000 simulated individuals across 1,912 STRUCTURE runs. On average, ancestry coefficients only deviated by ${\bar{Q}}_{\text{wild}}$ ± 0.0019 (range = 0.0002–0.0155). This indicated that ancestry coefficients were relatively stable and adequate point estimates. The average *Q*_wild_ for white‐tailed deer sampled from free‐ranging populations, excluding chronic wasting disease cases, was 0.913. Values for *Q*_wild_ ranged from 0.026 to 0.996 (Figure 4a). While wild ancestry was high among all subregions, average ancestry to the wild population varied (Table 1). Subregion 3 had the lowest mean *Q*_wild_ (0.800), while subregion 4 had the highest (0.947). Two deer from subregion 3 were found with ear tags, although public testimony suggested these to be wild deer tagged as fawns. Because of this public testimony, these deer were excluded from the generation of the captive reference cluster and they were considered as free‐ranging samples despite having ear tags. Ancestry coefficients were mixed, which may suggest both wild and captive ancestry (*Q*_wild_ = 0.328 and 0.588).

The average *Q*_wild_ for white‐tailed deer sampled directly from or thought to originate from captive facilities was 0.094. Values for *Q*_wild_ ranged from 0.005 to 0.951 for captive herds (Figure 4b). Only two captive deer had *Q*_wild_ > 0.500, and only a single captive deer from the Pennsylvania State University research herd had substantial wild ancestry (*Q*_wild_ = 0.951). Pedigree records showed that this deer was a habituated female that was legally removed from the wild. A free‐ranging deer from northeastern Pennsylvania was believed to have originated from a captive herd based on the identification tag found with the deer. Our results support this designation, with a *Q*_wild_ = 0.024.

### 3.3 Population assignment

We were able to correctly assign 96.9% of all simulated individuals to their respective reference populations using the Rannala & Mountain (1997) method. The majority of both wild (*n* = 1,754; 95.64%) and captive deer (*n* = 48; 94.12%) had the highest likelihood of assigning to their respective reference clusters. The average *A*_wild_ for wild deer was 0.951 and ranged from 0.003 to 1.000 (Figure 4c). We failed to reject both hypotheses (captive and wild origin) for 135 free‐ranging samples (7.36%). For the two tagged fawns from subregion 3, one assigned to the wild cluster (*A*_wild_ = 0.994, *p*_wild_ *= *.880, *p*_captive_ = .021), while the other assigned to both the captive and wild clusters (*A*_wild_ = 0.412, *p*_wild_ *= *.796, *p*_captive_ *= *.152).

The average *A*_wild_ for captive deer was 0.077 and ranged from 0.000 to 1.000 (Figure 4d). We failed to reject the hypothesis of captive origin for 48 captive samples (94.12%) but were only able to reject the hypothesis of wild origin for a single sample. The habituated female in the Pennsylvania State University captive herd assigned to the wild reference cluster (*A*_wild_ = 1.000, *p*_wild_ *= *.880, *p*_captive_ *< *.001). The free‐ranging deer from northeastern Pennsylvania, believed to have originated from a captive herd, assigned to the captive reference cluster (*A*_captive_ = 0.992), but we were unable to reject the hypothesis of wild origin (*p*_wild_ = .753).

### 3.4 Infected deer

The average *Q*_wild_ for chronic wasting disease infected deer was 0.911. All but two of the 27 samples had a *Q*_wild_ > 0.900, indicating wild ancestry (Table S1). One deer from subregion 3 had *Q*_wild_ < 0.500, while another from this subregion had *Q*_wild_ < 0.100. Similarly, average *A*_wild_ was high for all infected samples (0.933), with only the two samples from subregion 3 assigning to captive origin (*A*_wild_ = 0.235, *p*_captive_ *= *.090 and *A*_wild_ = 0.007, *p*_captive_ *= *.239, respectively; Table S1). This indicates possible captive origin and ancestry for two infected deer from subregion 3, although we were also unable to reject the hypothesis of wild origin for these deer (*p*_wild_ > .05). Both hypotheses (captive and wild origin) were rejected for one infected deer from subregion 2 (*p*_wild_ *= *.007, *p*_captive_ < .001), but ancestry and assignment scores indicate that wild ancestry and origin are more likely for this individual (*Q*_wild_ = 0.941, *A*_wild_ = 1.000; Table S1). All remaining infected deer only assigned to the wild reference cluster (*A*_wild_ > 0.900, *p*_wild_ > .05, *p*_captive_ ≤ .05).

## 4 DISCUSSION

### 4.1 Effects of captive egression

Unintentional or intentional release of diseased individuals from infected captive facilities may represent a potential mode of disease introduction into naïve wild populations. Recent outbreaks of chronic wasting disease in captive populations related to the movement of infected cervids highlight the potential role human‐mediated dispersal plays in establishing new outbreaks (Kahn et al., 2004; Kim et al., 2005; Sohn et al., 2002). Observed outbreaks in closely associated free‐ranging and captive populations have led managers to suspect a relationship between disease occurrences in these populations (Schuler et al., 2016). Although correlation was speculated in previous outbreaks, our results demonstrate that captive egression may have contributed to a novel chronic wasting disease outbreak in a county in subregion 3 where the disease was undetected in wild populations prior to 2017 and that was geographically separated from previously detected disease foci. Ancestry coefficients and assignment scores for two deer sampled in this county were higher for the captive reference population when compared to the wild (Table S1). We were unable to reject the hypothesis of captive origin for these individuals as well (*p*_captive_ > .05). These results indicate that these deer may have sourced from or share ancestry with captive populations in this area. We cannot conclusively link these cases back to infected cervid facilities using this method, but the geographic proximity of these individuals to captive facilities where chronic wasting disease was detected previously relative to the nearest cases in wild populations (>50 km) may indicate a possible relationship (Figure 1).

The frequency and extent of captive egression into wild populations is a highly debated issue. The average ancestry proportions and assignment scores of free‐ranging deer to the wild reference population were greater than 0.900 in both cases. This indicates that gene flow between captive and wild populations is low and that captive egression is likely limited. The rate of captive egression may not be spatially uniform, however, as the average ancestry and assignment scores varied among subregions (Table 1). The inferred rates of ancestry to captive populations and assignment to the captive reference cluster were highest in subregion 3, which is also where the two cases with high likelihood of captive ancestry and assignment occurred. This could indicate that captive egression and admixture are greater in this subregion. Carrollo (2016) predicted that subregion 3 would have a lower risk of chronic wasting disease establishment relative to other areas in Pennsylvania based on landscape factors, wild deer densities, and the operational practices of captive cervid facilities (stocking densities and translocations). Our results indicate that incorporating the potential influence of captive egression, which was not available in previous risk assessments, may improve such models. Captive ancestry and assignment were lowest in subregion 4, an area that has a moratorium on cervid farming.

It is important to recognize that captive egression represents only one potential avenue for captive‐wild interactions and should be taken as a minimum estimate of transmission risk from captive facilities. Interactions between wild and captive deer may occur along fences (VerCauteren et al., 2007a, 2007b) and represent another route of transmission that would remain undetected using genetic data. These interactions may also be a mechanism for the transmission of chronic wasting disease from wild populations to captive populations as well. Disease transmission risk may also be modulated by anthropogenic considerations, such as farm stocking densities, on‐site management practices, and the movement of deer among facilities (Brooks & Jayarao, 2008; Carrollo, 2016). Movements between facilities, in particular, may pose a substantial risk for captive‐mediated diffusion of chronic wasting disease, and other diseases, in wild populations (Gerhold & Hickling, 2016). Contact tracing based on 5,269 movement records demonstrates that approximately 62% of deer farms in this study region have either transferred or received at least one deer over a 7.68‐year period (Rorres et al., 2017). Approximately 31% of all translocations occurred over distance greater than 50 miles. This high rate of human‐mediated movement, when considered jointly with the potential for captive egression, represents a significant avenue for the introduction of chronic wasting disease into novel areas.

### 4.2 Performance of assignment tests

The utilization of simulated reference clusters as a basis for assignment may allow for the evaluation of captive egression in situations where it is impossible to sample a large number of potential source populations. We recognize that the rate of captive egression reported in this study was based on a limited number of samples. While the use of reference clusters partially alleviates this source of bias, a relatively large degree of variability was still observed among captive facilities (Figure 2). Sample sizes were often limited for these facilities, which may contribute to the observed variation among captive samples. Our samples were likely representative of the genetic stock of these facilities, however, as most captive cervid facilities in the study region maintain populations of 20 deer or less (Rorres et al., 2017). Operational practices can vary considerably among captive facilities (Brooks & Jayarao, 2008), which may also influence the degree of variation in herd separation observed between other captive and wild samples. Additional samples from other facilities may help to quantify the degree of genetic variation among captive herds, although obtaining samples from these facilities can be difficult. Nevertheless, the results of the ordination and assignment analyses show that it was possible to distinguish between wild and captive origin despite small sample sizes and low genetic differentiation (Figures 2 and 3). Additionally, we were able to assign successfully a wild female that was legally kept at a captive cervid research facility and a suspected escapee in wild populations back to their respective origin with high confidence. Our results were also commensurate with public testimony for two illegally tagged deer. While we were unable to reject the hypothesis of captive origin for one, ancestry and assignment scores suggested they were admixed. This indicates that a wild origin is still possible, although they may share ancestry to captive populations. These findings highlight the utility and efficacy of the applied genetic assignment methods in determining captive and wild origins. Loci with unique alleles that are specific to wild or captive sources may improve the power to formally test hypotheses regarding origin compared with frequency‐based methods used here. This may require genomic approaches to test numerous loci for such alleles. Nevertheless, the observed variation in ancestry and assignment scores provided a method for evaluating the relative likelihood of these deer sourcing from captive and wild populations.

### 4.3 Alternative disease establishment scenarios

Our results highlight the possibility of rare egression events from captive herds. It is plausible that egression may facilitate the spread of chronic wasting disease into free‐ranging populations if an individual is infected prior to egression. It is important to note that we cannot explicitly link individuals with captive ancestry or origin to infected facilities, so it is not possible to establish time of infection. Deer of captive origin may also contract chronic wasting disease following egression. Disease surveillance efforts were established in the area of this novel outbreak in 2014 due to detection of chronic wasting disease in captive herds. Despite ongoing surveillance, cases in the free‐ranging population were not detected until 2017 in this area. Given the time period between the initiation of targeted surveillance and detection of infected individuals in the wild and the high assignment and ancestry scores to the captive cluster for the cases in our study, infection prior to egression remains a plausible hypothesis regarding origin of these cases. Further surveillance is necessary to establish the extent of this current outbreak and prevalence within both free‐ranging and captive populations. This information, along with continued evaluation of the origin of novel infections, may provide additional insights into the relative contribution of captive and wild populations to the current outbreak.

Contact in the form of dispersal still represents an important potential pathway for the spread of chronic wasting disease in this region. All infected deer from subregions 2 and 4 were characterized by a high proportion of wild ancestry and assignment and were sampled in close geographic proximity in a single ecophysiographic province. This indicates a sustained infection in wild populations in this area. The only infected deer that assigned to and shared significant ancestry with the wild population from subregion 3 was found directly adjacent to the border between the subregions (<1 km away), which suggests it is likely associated with this outbreak as well. Wild white‐tailed deer are characterized by high dispersal rates (Long, Diefenbach, Rosenberry, Wallingford, & Grund, 2005; Lutz, Diefenbach, & Rosenberry, 2015), and dispersal away from infection foci may facilitate the diffusion of chronic wasting disease across landscapes (Evans et al., 2016; Hefley et al., 2017). While long‐distance dispersal events do occur and have been linked to spread of chronic wasting disease in other regions (Green et al., 2014), the novel outbreak in subregion 3 is in a separate ecophysiographic province and is separated from the larger outbreak in subregions 2 and 4 by a major topographic escarpment. Ridges are features that influence the dispersal patterns of white‐tailed deer in this region (Long, Diefenbach, Wallingford, & Rosenberry, 2010). Previous studies have demonstrated that landscape features that were resistant to deer movement and gene flow influence the prevalence and diffusion of chronic wasting disease (Blanchong et al., 2008; Hefley et al., 2017). Therefore, outbreaks in separate ecophysiographic provinces may have different origins. Landscape boundaries are permeable to deer movement and gene flow, however (Kelly et al., 2014; Robinson, Samuel, Lopez, & Shelton, 2012), so natural dispersal may also influence the epizootiology of chronic wasting disease in spatially distinct localities. Landscape genetic analyses have been used to predict movement‐mediated transmission patterns (Paquette, Talbot, Garant, Mainguy, & Pelletier, 2014) and may be useful in further evaluating the transmission risk wild sources relative to captive sources assessed here.

## 5 CONCLUSIONS

Understanding the source of disease outbreaks is an important goal of wildlife epizootiology studies. Assignment algorithms can be beneficial in identifying the putative source of novel infections, but traditional inference can be influenced by the presence of unsampled populations. By making use of differences in genetic variability within and among deer sampling units, we were able to create reference populations that allowed for initial evaluations of the ancestry and assignment of white‐tailed deer to captive and wild populations. Using this approach, we were able to discern the potential occurrence of captive egression. While this is seemingly rare, the detection of infected individuals with captive ancestry suggests that egression may contribute to the spread of chronic wasting disease within this region. The relatively few number of captive facilities sampled and low genetic divergence observed among wild populations highlighted the potential utility of this technique in situations that would typically lead to ambiguous results. Given this, similar methods may prove a useful tool for assessing the origin of novel disease cases in other systems as well. Incorporating samples from other captive facilities may lead to a better representation of the observed genetic variance in reference populations and further clarify the extent of egression and its influence on the epizootiology of chronic wasting disease. Continued disease surveillance and genetic analyses may also elucidate the relative risk of wild and captive sources of infection.

## CONFLICT OF INTEREST

None declared.

## Data Availability

Data for this study will be available at the Zenodo repository following a one‐year embargo period from the date of publication: ://doi.org/10.5281/zenodo.3530546.
