# Supplementary material for: Can genetic assignment tests provide insight on the influence of captive egression on the epizootiology of chronic wasting disease?
Source: Evol Appl. 2019 Dec 9;13(4):715–26. doi: 10.1111/eva.12895 (PMC7086050; doi:10.1111/eva.12895)
Supplement: Supplementary file 1 [file EVA-13-715-s001.docx]

**Appendix A.** Description and results of quality control procedures used to evaluate the quality and reproducibility of empirical microsatellite genotypes collected from white-tailed deer (*Odocoileus virginianus*) populations in the Mid-Atlantic region of the United States.

**Methods**

We reanalyzed a total of 179 samples (approximately 10% of the data) to ensure reproducibility of results. For null allele estimates and evaluation of equilibrium assumptions, we elected to exclude captive samples because sample sizes were small and populations were enclosed, which may predispose them to natural deviations from expectation. We estimated the frequency of null alleles in the dataset using the FreeNA algorithm (Chapuis & Estoup, 2007). Null alleles were estimated in the total dataset and for each free-ranging population with a sample size ≥ 25 individuals. Corrected F_ST_ values were compared to uncorrected F_ST_ values to determine the influence of observed null alleles on estimates of population structure. The program Genepop (version 4.6; Raymond & Rousset, 1995; Rousset, 2008) was used to evaluate deviations from Hardy-Weinberg and linkage expectations using default Markov Chain Monte Carlo parameters (10,000 dememorizations, 1000 batches, 10,000 iterations per batch). Significance was assessed using the Holm-Bonferroni procedure for multiple comparisons (Holm, 1979; Rice, 1989).

**RESULTS**

We were successfully able to genotype all individuals across 11 loci in >99% of cases. No individuals were missing genotypes at more than two loci. The estimated genotyping error rate did not exceed 0.8% for all loci. The average frequency of null alleles across all loci was 3.1% and no single locus exceeded 6.6%. Significant null allele frequencies (>10% frequency) were detected in 3 locus by populations comparisons (out of 231) and never exceeded 12.4%. There was strong collinearity between F_ST_ estimates uncorrected for null alleles and those that were corrected (*r* >0.99, maximum difference = 0.002, mean difference < 0.001), indicating that the observed frequency of null alleles did not produce an appreciable effect on population genetics estimates. Deviations from Hardy-Weinberg assumptions occurred in 3.5% of loci by population comparisons, with no single locus exceeding deviations in > 3 out of 21 populations. Linkage was found in one pair of loci in a single sampling unit out of 1155 possible combinations (<0.1%). These results suggest that genotyping errors were rare and not expected to significantly influence assignment tests or estimates of genetic structure, so we elected to include all individuals and loci in subsequent analyses.

**LITERATURE CITED**

Chapuis, M.-P., & Estoup, A. (2007). Microsatellite null alleles and estimation of population differentiation. *Molecular Biology and Evolution*, *24*(3), 621–631. https://doi.org/10.1093/molbev/msl191

Raymond, M., & Rousset, F. (1995). GENEPOP (version 1.2): Population genetics software for exact tests and ecumenicism. *Journal of Heredity*, *86*(3), 248–249.

Rousset, F. (2008). Genepop’007: A complete re-implementation of the genepop software for Windows and Linux. *Molecular Ecology Resources*, *8*(1), 103–106. https://doi.org/10.1111/j.1471-8286.2007.01931.x

Holm S. 1979. A simple sequentially rejective multiple test procedure. *Scandinavian Journal of Statistics,* *6*, 65–70.

Rice, W. 1989. Analyzing tables of statistical tests. *Evolution*, *43*, 223–225.
