# Supplementary material for: Can genetic assignment tests provide insight on the influence of captive egression on the epizootiology of chronic wasting disease?
Source: Evol Appl. 2019 Dec 9;13(4):715–26. doi: 10.1111/eva.12895 (PMC7086050; doi:10.1111/eva.12895)
Supplement: Supplementary file 2 [file EVA-13-715-s002.docx]

Table S1. Ancestry coefficients (Q), assignment scores (A), and assignment probabilities (*P*) for 27 white-tailed deer infected with chronic wasting disease from the Mid-Atlantic region of the United States.

| **Source** | **Q_wild_** | **Q_captive_** | **A_wild_** | **A_captive_** | ***P*_wild_** | ***P*_captive_** |
| --- | --- | --- | --- | --- | --- | --- |
| 2 | 0.985 | 0.015 | 1.000 | 0.000 | 0.159 | 0.000 |
| 2 | 0.977 | 0.024 | 1.000 | 0.000 | 0.322 | 0.000 |
| 2 | 0.953 | 0.047 | 0.999 | 0.001 | 0.405 | 0.001 |
| 2 | 0.925 | 0.075 | 0.999 | 0.001 | 0.730 | 0.002 |
| 2 | 0.972 | 0.028 | 1.000 | 0.000 | 0.084 | 0.000 |
| 2 | 0.987 | 0.013 | 1.000 | 0.000 | 0.083 | 0.000 |
| 2 | 0.941 | 0.059 | 1.000 | 0.000 | 0.007 | 0.000 |
| 2 | 0.986 | 0.015 | 1.000 | 0.000 | 0.786 | 0.000 |
| 2 | 0.990 | 0.010 | 1.000 | 0.000 | 0.170 | 0.000 |
| 2 | 0.988 | 0.012 | 1.000 | 0.000 | 0.743 | 0.000 |
| 2 | 0.971 | 0.029 | 1.000 | 0.000 | 0.772 | 0.001 |
| 2 | 0.962 | 0.038 | 1.000 | 0.000 | 0.308 | 0.000 |
| 2 | 0.975 | 0.026 | 1.000 | 0.000 | 0.639 | 0.000 |
| 2 | 0.989 | 0.011 | 1.000 | 0.000 | 0.400 | 0.000 |
| 2 | 0.980 | 0.020 | 1.000 | 0.000 | 0.269 | 0.000 |
| 2 | 0.910 | 0.090 | 0.958 | 0.042 | 0.590 | 0.013 |
| 2 | 0.995 | 0.005 | 1.000 | 0.000 | 0.072 | 0.000 |
| 2 | 0.988 | 0.012 | 1.000 | 0.000 | 0.147 | 0.000 |
| 2 | 0.922 | 0.078 | 1.000 | 0.000 | 0.171 | 0.000 |
| 2 | 0.967 | 0.033 | 0.993 | 0.007 | 0.125 | 0.000 |
| 2 | 0.981 | 0.019 | 1.000 | 0.000 | 0.262 | 0.000 |
| 3 | 0.976 | 0.024 | 1.000 | 0.000 | 0.274 | 0.000 |
| 3 | 0.040 | 0.960 | 0.007 | 0.993 | 0.413 | 0.239 |
| 3 | 0.309 | 0.691 | 0.235 | 0.765 | 0.564 | 0.090 |
| 4 | 0.976 | 0.024 | 1.000 | 0.000 | 0.322 | 0.000 |
| 4 | 0.975 | 0.025 | 0.997 | 0.003 | 0.786 | 0.008 |
| 4 | 0.986 | 0.139 | 1.000 | 0.000 | 0.703 | 0.000 |

*Note*: Deer were stratified by subregion (Source). Ancestry coefficients, assignment scores, and assignment probabilities are listed with subscripts specifying the simulated reference population.
